# Probiotics, Prebiotics, and Synbiotics in Pigs and Poultry: A Review of Gut Health, Performance, and Environmental Outcomes

**DOI:** 10.3390/vetsci12111054

**Published:** 2025-11-02

**Authors:** David Atuahene, Bernard Abeiku Sam, Frank Idan, Shaikh Sumayya Sana, Renáta Knop, Tejas Suthar, Harsh Kumar, Ayaz Mukarram Shaikh

**Affiliations:** 1 School of Agriculture and Veterinary Medicine, University of Turin, 10095 Grugliasco, Turin, Italy; 2 Department of Animal Sciences, University of Arkansas, Fayetteville, AR 72701, USA; basam@uark.edu; 3 Department of Animal Sciences, Faculty of Agriculture, College of Agriculture and Natural Resources, Kwame Nkrumah University of Science and Technology, Kumasi AK000-AK911, Ghana; frank.idan@knust.edu.gh; 4 Faculty of Agriculture, Food Science & Environmental Management, Institute of Animal Science, University of Debrecen, Böszörményi út 138, 4032 Debrecen, Hungary; sumayya.sana@agr.unideb.hu (S.S.S.); dr.knop.renata@agr.unideb.hu (R.K.); 5 Independent Researcher, Chicago, IL 60616, USA; suthartejas2525@gmail.com; 6 Faculty of Materials Science and Technology, VSB-Technical University of Ostrava, 70800 Ostrava-Poruba, Czech Republic; microharshs@gmail.com; 7 Department of Food Science and Technology, Graphic Era (Deemed to be University), Dehradun 248002, Uttarakhand, India; 8 Faculty of Agriculture, Food Science, and Environmental Management, Institute of Food Science, University of Debrecen, Böszörményi út 138, 4032 Debrecen, Hungary

**Keywords:** gut health, nutritional efficiency, monogastric livestock, animal welfare, environmental sustainability

## Abstract

**Simple Summary:**

This review article emphasizes the significance of gut health for monogastric livestock, precisely pigs and poultry. A reasonably operating gastrointestinal system facilitates digestion, nutrition absorption, immunological functionality, and infection resistance, therefore enhancing growth and feed efficiency. In disparity, illnesses, inadequate meals, and stress hinder absorption, elevate veterinary and husbandry expenses, and exacerbate environmental impacts through nutrient losses and emissions (e.g., CH_4_, NH_3_).This article consolidates evidence that nutritional modulation specifically probiotics, prebiotics, and targeted feed additives can cultivate a stable, advantageous microbiota increase microbial diversity, fortify the mucosal barrier, and elevate short-chain fatty acids. These changes enhance nutrient absorption, growth velocity, and feed efficiency, while diminishing pathogen burden and the necessity for therapeutic or prophylactic antibiotics, in accordance with stewardship objectives. The review demonstrates that gut-focused feeding practices enhance production, animal welfare, and profitability while concurrently decreasing waste and pollution. Which, incorporated into lifecycle management, these methodologies provide an interconnected course from biological processes to systemic sustainability in pig and poultry production.

**Abstract:**

The cardinal Physiology of Gut Health in monogastric animals such as swine and poultry is vital. It is critical for digestive efficiency, immune status, and production levels. This system is related not only to the digestion and absorption of nutrients from feed ingredients contributing to growth and feed utilization efficiency but also to having a strategic microbiota that supports immunity and pathogen resistance, as well as metabolic support. Gut disease, for example, bacterial, viral, or parasitic infection, diet, or stress, can reduce nutrient digestion and absorption. They can also suppress the immune system and render patients more prone to disease. These are efficiency degradations and increase veterinary and husbandry costs. In addition, nutrient absorption because of deteriorated gut health can affect the environment in different ways: removal of nutrients through leaching and the release of gases (including CH_4_ and NH_4_). These pressures have led to a focus on the gut in animal research to improve the welfare of animals and ensure sustainable practices in animal production. Recent studies have included the use of probiotics, prebiotics, and other feed additives to enhance the positive effects of the gut microbiota. These are also intervention points to increase nutrient absorption and animal well-being, in turn sustainability. Such approaches are expected to promote a stable microbial community with less dependence on the use of antibiotics, less waste generation, and less environmental impact from animal farming. This review provides a critical evaluation of the current literature on gut health in monogastric livestock, with pigs and poultry as the principal focus. We also considered the impact of gut health on production efficiency and Environmental sustainability. Current progress in nutritional modulation of gut health for increased productivity, enhanced animal welfare, and better profitability are presented. Gut-related biological mechanisms are linked to practical nutritional strategies, and subsequently to animal welfare, production efficiency, and environmental effects, offering a coherent concept for moving from mechanism to system-level sustainability.

## 1. Introduction

Gut function directly influences the growth, feed efficiency, and disease resilience of monogastric species (pigs and poultry). Diet-induced changes in the microbiota, barrier integrity, short-chain fatty acid (SCFA) signaling, and immune priming can be modulated by targeted nutrition (e.g., probiotics, prebiotics, and synbiotics), which may mitigate the impact of post-weaning diarrhea. It can also enhance the Feed Conversion Ratio (FCR) and reduce nitrogen loss. Pigs are a foundation of global livestock systems, providing nearly half the world’s meat and supporting food security, trade, and rural livelihoods throughout Asia, Europe, and the Americas [1]. More than production, pigs are crucial to biomedical research due to their anatomic and physiologic resemblance to humans, which contributes to the creation of translational models in cardiometabolic, gastrointestinal, and immunological conditions [2]. The significance of these is epitomized by the rapid progress in xenotransplantation, specifically gene-edited pig organ transplantation trials. Recent clinical-level kidney xeno-grafts open a path for rigorously safe, regulated addressing of organ shortages [3,4]. In summary, the dual agricultural and biomedical functions of pigs magnify the stakes in gut health interventions, as nutritional and microbial approaches that enhance productivity may also reveal human-relevant mechanisms and clinical innovations. Changes in gut health significantly impact the quality of life in monogastric animals. The gastrointestinal (GI) tract is a key process that regulates nutrient digestion and absorption, and the function and structure of the GI tract can influence the rate of growth, feed efficiency, and production [5,6]. The GI tract serves not only as the site of digestion but also as a habitat for a myriad of microbial communities, which play critical roles in immune system development and function, protection against infections, and host metabolism [7,8]. This research explores the connection between gastrointestinal health, animal welfare, and an animal’s capacity to cope with environmental fluctuations. Pathogenic infections, malnutrition, and stress may damage gut health, leading to malabsorption of nutrients, immune dysfunction, and an increased risk of disease [9,10,11]. These challenges reduce production efficiency, increase antibiotic use, and raise veterinary costs, all of which contribute to lower profitability for the farmer [5,6,12].

There are important links between GI health and the welfare, productivity, and environmental intensity of livestock production [13,14]. Poor gut health is often associated with leaky gut syndrome, which results in poor nutrient absorption and feed digestion. Thus, the status of animals progressively deteriorates [15,16,17]. This inefficiency is reflected in the excretion of nitrogen, phosphorus, and other waste products, which negatively impact the environment [18,19]. Additionally, an imbalance in the gut microbiota increases ammonia and methane production—two main greenhouse gases [19,20,21]. These are linked to FCRs, which indicate the environmental impacts of livestock production. A balanced diet that promotes optimal gut health enhances nutrient uptake and waste production, while regulating processes to achieve sustainable agriculture [6]. An improved FCR and lower gas emissions result in higher income for producers, enhanced global warming mitigation, and reduced agricultural pollution [22].

Probiotics, prebiotics, and other dietary interventions to modulate gut microbiota are the most promising new approaches for gut health management. Several studies have noted that probiotics improve the barrier function of the gastrointestinal tract, influence immune modulation, and increase the number of beneficial microorganisms in swine and poultry [17,23,24,25]. Prebiotics are nondigestible food ingredients that promote the growth and/or activity of certain bacteria that are beneficial to the gut microbiota and have a positive effect on the gut microbiota. The synergistic use of these interventions offers an integrated strategy for animal gut health management with enhanced nutrient utilization, growth performance, and immunity [26,27]. In addition, such methods might contribute to a decrease in antimicrobial resistance (AMR) by avoiding or minimizing antibiotic use, and as a result, the selective pressure for the formation of resistant strains [27]. In the present review, recent advances in such strategies are discussed, focusing on concentrations in pigs and poultry, and possibilities for enhancing system efficiency and livestock farming sustainability are considered. This review comprehensively summarizes the beneficial effects of enhanced gut health in animals and environmental systems. Specifically, we focused on evidence for the effects of the barrier of probiotics/prebiotics on the integrity of the immune system, nutrient utilization, and nitrogen/phosphate (N/P) emissions in pigs and poultry. This review is structured to follow a cause–effect sequence: (i) we introduce nutritional strategies that alter the gut milieu; (ii) we describe the microbial and mucosal bases of the products of these strategies; (iii) we integrate information to demonstrate how the underlying microbial and mucosal mechanisms enhance nutrient digestion and absorption and animal performance; and (iv) we relate these techniques in production systems to potential consequences for animal welfare and the environment, and end with practical constraints and research needs for their delivery. To address this integrative model, we begin by reviewing the current knowledge of gut biology and selected nutritional strategies, then examine the extent to which these strategies influence nutrient absorption and animal performance, and consider implications for animal welfare and environmental outcomes.

## 2. Literature Search & Evidence Grading

### 2.1. Search Strategy

We searched PubMed, Web of Science, Scopus, and CAB Abstracts (January 2005–August 2025) using combinations of: (“probiotic” OR “prebiotic” OR “synbiotic” OR “postbiotic”) AND (pig* OR swine OR broiler* OR poultry OR chicken*) AND (gut OR intestine OR microbiota OR “feed conversion” OR FCR OR diarrhea OR ammonia).

### 2.2. Eligibility Criteria (PICOS)

- Population: Monogastric food-producing species limited to pigs (suckling, weaned, grower–finisher, and sows) and poultry (broiler and layer chickens). Studies focusing exclusively on ruminants or non-target species were excluded from the review.
- Intervention: Dietary administration (feed or water) of probiotics (live microorganisms), prebiotics (selectively utilized substrates), or synbiotics (combined probiotic–prebiotic). Postbiotic studies were included only when the mechanistic endpoints were directly linked to in vivo outcomes in pigs or poultry.
- Comparator: Placebo/basal diet, standard management practices, or active controls (e.g., antibiotic growth promoters, alternative feed additives). Challenge models with pathogen exposure were eligible if a concurrent control group was present.
- Outcomes: At least one primary in vivo outcome relevant to health or productivity was required, including one or more of the following: average daily gain (ADG), body weight (BW), feed intake, FCR, morbidity (e.g., diarrhea incidence) or mortality, nutrient digestibility/retention, nitrogen or phosphorus excretion, and environmental emission proxies (e.g., ammonia). Mechanistic and physiological endpoints were included when measured alongside in vivo performance or health outcomes in pigs or poultry. These endpoints included gut barrier integrity, tight junction expression, mucin, immune markers such as IgA and cytokines, SCFA, microbiota composition and diversity, and pathogen load. Endpoints were also included when they were clearly linked to in vivo performance or health outcomes in pigs or poultry.
- Study design: In vivo randomized controlled trials (RCTs), controlled field trials, challenge trials with controls, and meta-analyses/systematic reviews. Relevant mechanistic studies were included only if they were linked to in vivo outcomes in the target species.

### 2.3. Additional Inclusion Parameters

Please refer to the Supplementary File for details.

## 3. Nutritional Strategies for Optimizing Gut Health

Diet composition has a direct impact on gut microbial profiles in monogastric animals, so having a balanced diet is crucial for the health and productivity of animals. Fibers, amino acids, and antioxidants have important functions in microbial growth and host-microbiota homeostasis [28]. Non-starch polysaccharides are substrates for gut microorganisms, and certain amino acids are used for protein synthesis in immune responses and gut repair [29]. Vitamins A, C, and E safeguard the gastrointestinal mucosa against oxidative stress and inflammation while favoring a beneficial gut microbiota, which is also required for digestion, absorption, and well-being of the host [30].

**Figure 1:**
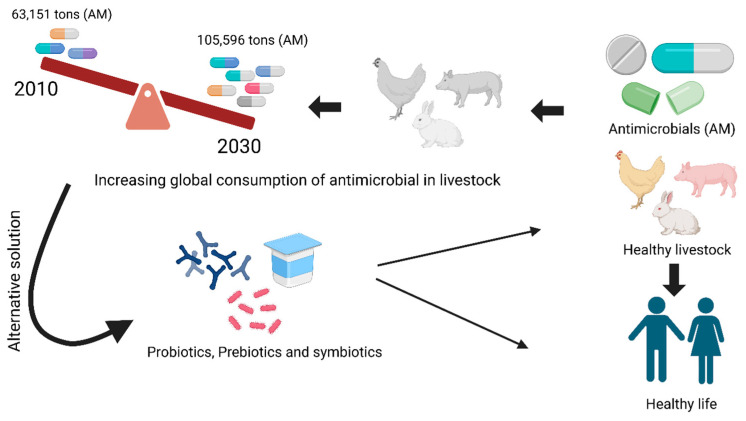
Probiotics as an organic Agent towards Livestock Health and Production in the Increasing Antibiotic Age. (Figure created with Biorender.com).

Much interest has been directed toward the use of feeding additives to enhance gut health. Prebiotics, probiotics, and synbiotics are effective in modulating the gut microbiota in monogastric animals [31,32,33]. Two of these, oligosaccharide and inulin, have been demonstrated to increase the number of beneficial bacteria and reinforce intestinal barrier function. Probiotics, live beneficial organisms, regulate the composition of the gut microbiome, prevent the proliferation of pathogenic microorganisms, and enhance gut lining integrity. Synbiotics are combinations of probiotics and prebiotics that support the growth and activity of probiotics, thus providing synbiotics that act synergistically to enhance gut function and host health. These functional ingredients enhance the efficiency of feed conversion, contribute to growth promotion in swine and poultry, and strengthen the immune system [34,35,36,37]. Probiotics are becoming new biotic agents for promoting animal health and production in the face of antibiotic resistance (Figure 1). Figure 1: Probiotics as livestock production and health enhancers in the era of increasing antibiotic use. (Figure created using Biorender.com; BioRender version 2.2). The global need for meat and milk is expected to continue to grow, but the use of antimicrobial drugs in animal production is expected to increase by 67% by 2030, mainly owing to intensified animal agriculture (Figure 1) [38,39,40].

However, rising antibiotic resistance has driven the adoption of probiotics as safer, sustainable alternatives [41,42]. Probiotics enhance digestive function, strengthen immunity, and improve growth performance, reducing antibiotic reliance while promoting better health and production efficiency [43,44,45]. This trend is in line with the expanding need for animal welfare and public health, because of the expansion of international food production. Fermentation products and natural compounds promote gastrointestinal health. SCFAs are mainly acetic, propionic, and butyric acids, produced by the fermentation of dietary fiber by gut microbiota. These SCFAs are utilized as sources of energy, modulate immune responses, and lower the pH of the gut, which inhibits the growth of pathogenic microorganisms [46,47,48,49]. Long-lasting good health was promoted in a murine model of sepsis by polyphenol-rich fermented products through the modulation of the gut microbiome and metabolites. Polyphenols and flavonoids possess antimicrobial, antioxidant, and anti-inflammatory activities, influence gut microbiota composition, attenuate oxidative stress, and improve intestinal barrier function. Supplementation with fermented products and natural compounds is an innovative feeding strategy that positively affects gut health, enhances the palatability of nutrients, decreases the use of antibiotic growth promoters, and benefits both animal health and environmental preservation. The main dietary factors and animal feed supplements that contribute to the enhancement of monogastric gut health are summarized in Table 1. It deals with the potential role of fiber and amino acids and the use of antioxidants, prebiotics, probiotics, synbiotics, and fermentation products in maintaining gut microbiota, digestion, and immune response. In addition, their effects on the performance (including feed to gain ratio, growth, and immunity) of livestock (Table 1). Having described the major dietary and animal feed supplements that can control the gut community, the following section will discuss the physiological mechanisms wielded by these substrates, in particular by prebiotics and probiotics, to modulate microbiota composition, barrier function, and host immune signaling.

## 4. Probiotics and Prebiotics: Mechanisms of Action

**Figure 2:**
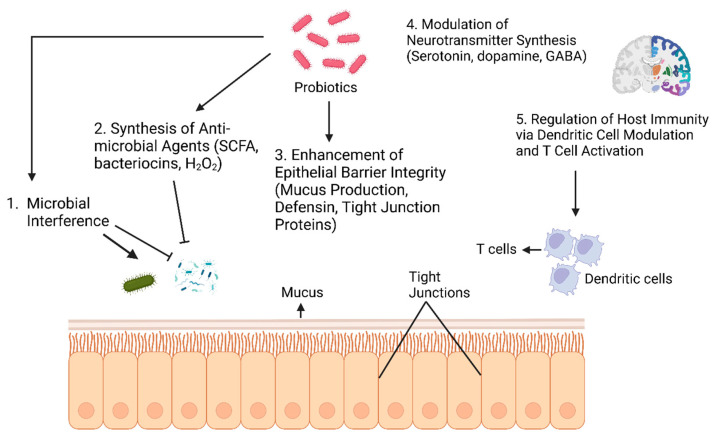
Mechanism of action of probiotics. (Figure created with Biorender.com).

Probiotics are live, non-pathogenic microorganisms that confer health benefits when administered in adequate amounts [58,59,60,61]. They enhance gut health primarily by modulating the gastrointestinal microbiota through the competitive exclusion of pathogens and production of antimicrobial compounds, such as lactic acid and bacteriocins. Additionally, they strengthen intestinal barrier integrity by promoting mucus secretion and the expression of tight junction proteins. They also provide immunomodulation by enhancing cytokine and immunoglobulin production [62,63,64]. Probiotics exert their beneficial effects through various mechanisms of action (Figure 2).

Probiotics compete with pathogens for nutrients and binding sites, making it more difficult for harmful microbes to survive or attach to the gut lining (Figure 2). They produce substances that inhibit pathogen growth, boost mucus production, and increase tight junction protein expression, all of which strengthen the intestinal barrier and prevent the entry of pathogens into the bloodstream. Probiotics also support immune function by influencing dendritic cell maturation and T cell proliferation. In addition, they affect neurotransmitter production, including serotonin, dopamine, and gamma-aminobutyric acid (GABA).

Many probiotic strains have been reported to produce diverse results because they function differently. The probiotics that have been extensively studied are *Lactobacillus* and *Bifidobacterium*, which improve the gut health of pigs and poultry. *Lactobacillus* is a lactic acid–producing bacterium that helps maintain low colonic pH, thereby inhibiting the growth of pathogenic microorganisms. It also promotes the production of short-chain fatty acids, which serve as energy substrates, and supports the integrity of the intestinal epithelial barrier [65,66,67]. *Bifidobacterium* species can ferment carbohydrates to produce SCFAs and other metabolites, which enhance the rate of increase in healthy gut bacteria. These probiotics enhance the immune function by stimulating immunoglobulin synthesis. In addition, they regulate the inflammatory responses. *Lactobacillus* and *Bifidobacterium* strains have been reported to improve the gut health of pigs and poultry in terms of growth rate, nutrient utilization, and immune status. They are important because they can control the machinery of the gut microbiota and the immune system. This control helps maintain gut health. Additionally, it reduces reliance on antibiotics in animal production [67].

**Figure 3:**
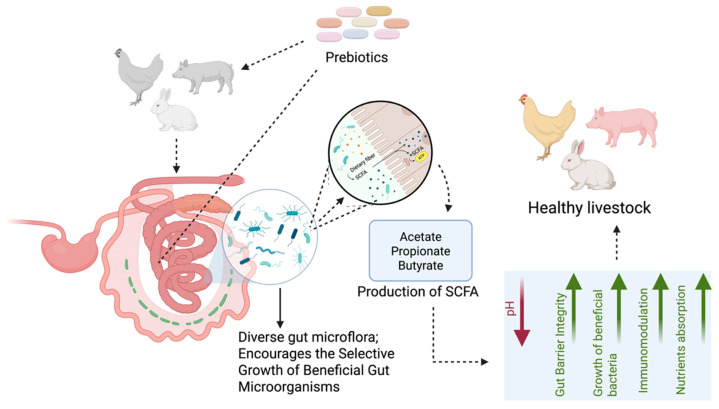
Mechanism of action of prebiotics (Figure created with Biorender.com).

Prebiotics are nondigestible food ingredients that selectively stimulate the growth and activity of beneficial bacteria in the gastrointestinal tract. Common prebiotics in livestock feeds include oligosaccharides, inulin, and fructooligosaccharides (FOS). These compounds resist upper GI tract digestion and reach the colon intact, where they undergo selective fermentation by beneficial microbiota, particularly *Bifidobacterium* and *Lactobacillus* [63,66,67,68,69] (Figure 3).

Prebiotics, such as fibers and oligosaccharides, pass through the upper digestive tract undigested and reach the colon (Figure 3). They are broken down by beneficial gut bacteria to produce SCFAs. SCFAs lower pH, which helps block harmful bacteria, strengthen the gut barrier, and support good bacterial growth. Prebiotics also help regulate immunity and improve mineral absorption, aiding overall intestinal and body health.

### Prebiotic Regulation Mechanisms

Mechanistically, inulin-type fructans and oligosaccharides are fermented by commensal bacteria to generate acetate, propionate, and butyrate. SCFAs signal through G protein-coupled receptors (GPR41/FFAR3 and GPR43/FFAR2) and act as histone deacetylase inhibitors to upregulate tight junction proteins (ZO-1 and occludin), mucin expression, and epithelial energy metabolism. These actions reinforce barrier integrity and lower luminal pH, thereby limiting enteropathogens relevant to post-weaning diarrhea and necrotic enteritis [70,71].

SCFAs and prebiotic-driven metabolites shape bile acid pools and antimicrobial activity while increasing IgA production and tempering nuclear factor-κB-mediated inflammation. In monogastrics, these pathways improve villus architecture, transporter expression, and nitrogen utilization, resulting in enhanced ADG and FCR. Yue Et Al. (2025) demonstrated that prebiotics, probiotics, symbiotics, and postbiotics act along complementary axes—microbial selection, metabolite signaling, and immune/barrier modulation—highlighting SCFA-centered barrier reinforcement as core mechanisms underpinning productivity gains and reduced antibiotic reliance [70,71].

It was recently demonstrated that 2-hydroxy-4-methylpentanoic acid (HMP) derived from Bacillus subtilis upregulates GADD45A and its downstream Wnt/β-catenin signaling. ZO-1 and occludin levels were also elevated, providing insight into how prebiotics and microbial approaches can be used to restore the barrier in monogastric livestock. *Lacticaseibacillus*-laden GABA signaling has also been associated with growth, antioxidative ability, and mucosal health. For example, prebiotics that promote GABA-producing lactobacilli may enhance the neuro-immune-epithelial advantage in monogastrics exposed to production stress [71].

SCFA generation is increased by prebiotics, which in turn reduces gut pH and discourages pathogen load, which also supplies energy substrates for colonocytes. Fermentation enhances gut barrier function, immunity, and mineral absorption. Reports have shown that prebiotics increase the levels of immunoglobulins, phagocyte activity, and disease resistance, and decrease the levels of inflammatory molecules in swine and poultry. Collectively, they are involved in enhanced feed efficiency, decreased environmental pollution by waste, and increased general productivity of livestock [62,72,73,74,75]. Supplementary Table S1 shows specific probiotic- or prebiotic-related effects on gut health, gut microbial composition, digestion, immunity, and, in some cases, nutrient use in monogastric animals. Elucidation of such mechanisms also explains the potential for synergy from the combination of substrates able to feed beneficial microbes (prebiotics) and live beneficial strains (probiotics): the next section explores evidence for combined (synbiotic) benefits upon microbiota stability and host performance.

## 5. Synergistic Effects of Probiotics and Prebiotics

Synbiotics, combinations of probiotics and prebiotics, are gaining attention for improving gut health in monogastric animals, such as pigs and poultry [76,77,78]. Probiotics are live microbes that help balance the gut microbiota, whereas prebiotics are substances that feed beneficial microbes [78]. Together, they work synergistically to diversify and stabilize the gut microbiota, supporting digestion, nutrient absorption, and immune functions. Synbiotics are especially valuable in livestock production, boosting productivity and reducing the need for antibiotics [79,80].

Scientific studies and case reports have shown that synbiotics can enhance the gut state and performance of animals. The initial findings indicate that synbiotics have the potential to increase microbial density within the gastrointestinal tract. This is significant because microbial content influences nutrient digestion and absorption [81,82]. Enhanced growth rates, improved FCRs, and overall health have been observed. These findings suggest that antibiotic use is associated with a low incidence of disease. Previous research has demonstrated that prebiotics can affect the production of SCFAs, which can be utilized by the host for energy production and contribute to reinforcing the gut barrier. Prebiotics have been shown to increase immunoglobulin and phagocytic indices, reduce inflammatory indices, and enhance disease immunity in pigs and poultry [75,83,84]. Furthermore, synbiotics improve the immune status of the gastrointestinal tract in animals, leading to a reduction in disease incidence and enhanced production of beneficial metabolites. Based on these studies, synbiotics are deemed to be suitable for promoting gut health and improving animal welfare. These changes in microbial genera due to the use of probiotics and prebiotics influence the immunological status, growth, and production traits in animals. One of the primary advantages is the enhancement of the FCR, which is crucial when determining the rate at which animals convert feed into tissue [85,86,87].

The gut microbiota is re-established through synbiotics, which are agents that promote nutrient consumption. This means that the animals need less feed to get as size first, so the FCRs standard is increasing. Preclinical and clinical research have shown that the administration of probiotics combined with prebiotics can improve the growth performance of pigs and poultry. Such supplementation increases the rate at which market weight is attained and decreases FCRs. Indeed, in porcine models, treatment with *Lactobacillus* spp. and inulin has been linked to increased BW gain and enhanced muscle growth [88,89,90]. Synbiotics have also been applied in poultry as feed enhancers to improve the rate of weight conversion. The reported gains in growth rate and feed efficiency have a direct profit impact on livestock farming enterprises by decreasing the cost of production and increasing income [91,92,93].

Synbiotics have been shown to affect the general health of animals. They also influence the disease threshold and, thus, overall productivity. Synbiotics are substances that increase the diversity and abundance of gut microbiota. They alter the balance of the microbial community, leading to a better gut barrier function. They also inhibit the colonization of hostile bacteria and reduce the probability of gastrointestinal infections [85,94]. A reduction in disease incidence has far-reaching positive consequences. One notable effect is the decline in antibiotic use in livestock. This decline is especially important in light of the global shift toward more sustainable agricultural methodologies. An animal model of potential synbiotics that enhance the immune system has been shown. In particular, these investigations demonstrated increased levels of circulating immunoglobulins and increased phagocytic activity. Such immunomodulation may play a role in immunity against some pathogens and lead to faster recovery when a person contracts infectious diseases [95,96].

Moreover, consumption of synbiotics has also been linked to diminished colonic inflammation, leading to better overall health. Thus, synbiotics could be exploited as a strategic option to reduce the environmental footprints related to animal farming. This is achieved via improved animal health, higher productivity levels, and reduced dependence on pharmaceuticals [73,97,98].

In addition to affecting growth and production parameters, synbiotics have also been reported to improve the quality of animal products, such as meat, eggs, and milk. For instance, in poultry, synbiotics have been linked to improved meat quality. Furthermore, synbiotics have been proven to decrease the levels of harmful substances in the gastrointestinal tract, such as ammonia and endotoxins, which are detrimental to the health of birds. This decrease frequently leads to animal products, such as meat and eggs, which have fewer chemical contaminants that threaten the health of people who consume them. In piglets, synbiotics positively influence carcass quality by increasing lean meat percentage and decreasing fat tissue. These improvements in product quality help make animal farming sustainable. In addition, they are accompanied by an increase in the nutritive value of the products and a decrease in waste [97,98,99].

Concurrently, the concurrent delivery of probiotics and prebiotics, the so-called synbiotics, represents an intriguing approach to further improve gut health and production in monogastric species. The ability of synbiotics to modulate the composition of the gut microbiota has been well established. Such modulation could potentially enhance nutrient utilization, immune system function, resistance to pathogenic attacks, feed conversion rate, and growth performance [85,94,98]. These results favor better soil management, decreased antibiotic usage, and enhanced animal welfare. The use of synbiotics results in additional improvements in animal-based goods and contributes to the well-being of both animals and consumers. Future studies should address the incorporation of synbiotics into animal production systems. Such investigations are necessary to build sustainable, holistic models for animals raised for food and other uses [97,98,99].

The changes in microbiota and barrier/immune functions that synbiotics use directly induce modifications in digestion and absorption. In Section 6, we outline the effects of these microbial and mucosal changes on nutrient utilization and feed conversion.

## 6. Impact on Nutrient Absorption and Utilization

The beneficial effect on gut health in monogastric animals, including swine and poultry is of paramount importance concerning the nutritional value of the feed [100,101]. The gut microbiota plays a crucial role in digestion, as it breaks down complex nutrients that the bloodstream cannot absorb directly. Beneficial colonization by bacterial species produces SCFAs, including butyrate. These SCFAs are also important to the host in terms of energy and gut barrier integrity. Moreover, healthy gut microbiota induces the expression of nutrient transporters in gut epithelial cells. This overexpression increases the translocation of amino acids, fatty acids, and glucose into the host cells. In summary, the above mechanisms enable greater solubilization, absorption, and utilization of nutrients, thereby supporting metabolic functions and promotion [48,102].

Gut health has a considerable effect on protein, fat, and carbohydrate metabolism in monogastric animals. The gastrointestinal status is especially important for protein metabolism, as the intestinal microflora is involved in the enzymatic degradation of large protein molecules to peptides and amino acids in the host organism. In addition, compounds that modify the composition of the intestinal microbiota, such as bioactive compounds, have an impact on protein digestibility. In fact, bifidobacteria and lactic acid bacteria, which are considered probiotics and prebiotics, are known to increase protein digestibility by increasing the number of bacteria that can synthesize proteolytic enzymes [101,103,104].

An increased rate of protein metabolism accounts for enhanced rates of growth and muscle accretion as proteins are oxidized for tissue synthesis. Similarly, lipid utilization is activated by gut microbiota, which can digest dietary lipids.

The fermentation of dietary fiber and nondigestible carbohydrates by intestinal microbiota results in the formation of SCFAs, which are involved in lipid metabolism [104,105,106]. Among them are several strains, including *Lactobacillus* species, which are known to be involved in lipid metabolism. They play a role in reducing fat storage and increasing fat use as fuel, leading to improved body composition. Gut health also favorably affects carbohydrate metabolism, because the role of the microbial consortium in digesting complex carbohydrates into simple sugars is well established. These sugars can then easily diffuse into the host tissues. In summary, improved gut health allows for better digestion of proteins, fats, and carbohydrates, which results in more efficient utilization of energy by animals and ultimately improves animal performance [48,101,103].

Enhanced gut health significantly enhances nutrient absorption and metabolic processes. Improved nutrient utilization is positively associated with augmented growth rates in swine and poultry species [107,108]. These include increased feed efficiency, a key indicator of the productivity of animals raised for food production. Enhanced gut health reduces the energy expenditure required for digestion, thereby allocating more energy to the growth processes. Consequently, this leads to an improved FCR, which is characterized by more rapid weight gain and reduced feed intake. For example, experimental studies have demonstrated that some identified probiotics, including *Lactobacillus* and *Bifidobacterium*, improve FCR by increasing the efficiency of dietary protein and energy utilization. In poultry, it has been reported that inulin or oligosaccharide supplementation in the diet increases nutrient solubility, which in turn escalates the feed intake and growth rates among birds [109,110,111]. An experiment involving broiler chickens demonstrated that supplementation with a synbiotic formulation containing both probiotics and prebiotics resulted in enhanced growth performance, specifically in terms of ADG and feed intake, compared to a control group. This finding indicates that enhanced gut function is a major driver of performance. Moreover, additional evidence suggests that improving gut health reduces the risk of gastrointestinal diseases, including diarrhea. This decrease in the prevalence of GIT disease is associated with better weight gain and feed conversion efficiency (FCE) [110,112,113]. In these cases, shortening of the digestive tract was affected by treatment with probiotics and prebiotics. The treatment was successful in improving the growth rates and other animal performance measures. In addition to feed conversion and growth efficiency, gut health is also important for enhancing an animal’s ability to withstand challenges, such as infectious agents or feed changes, regardless of species. These are important stressors that can have detrimental effects on growth performance if not controlled by good management of gut health.

Recent reports have shown that in swine, the stabilization of the gut microbiota, which is a vital part of gut health, reduces the adverse effects of stress on both production and growth performance [114,115]. The gut microbiota is essential for shaping the immune system. Stimulation of the immune system by gut microbes may play a role in decreasing inflammation and oxidative damage, both of which negatively affect cell proliferation. A number of porcine studies have indicated that the administration of probiotics can result in lower levels of cortisol, a marker of physiological stress, and improved immune function. The overall effect of these is better health and growth [116,117,118]. In poultry, a balanced gut microbiota is important for the host to resist diseases. This is achieved by reinforcing the barrier of the gut to avoid pathogen intrusion. As a result, the use of antibiotics was reduced. The less sick the animal, the less growth retardation it suffers, and the better the FCRs it tends to achieve [116,119,120,121]. Improvements in gut health, reduction in the prevalence of diseases, and more efficient nutrient assimilation and absorption are the means by which further productivity gains can be attained. This implies that to maximize the digestive efficiency of livestock and improve their overall performance parameters, adequate control of gut health is significant. Gut health is associated with workplace productivity in several research reports. As an example, a randomized controlled trial with porcine subjects showed that BW gain and FCR were increased in *Bifidobacterium* probiotic-treated groups when compared to controls. This probiotic supplementation seems to lead to the proliferation of beneficial gut bacteria and enables better catabolism of proteins and energy substrates [122,123]. The addition of prebiotic oligosaccharides to poultry diets improves nutrient digestion, particularly in the small intestinal region. This enhancement is reflected in the improved growth performance and feeding efficiency [124,125,126,127].

Together, these results confirm previous findings that the secret to maximum performance in birds is a healthy gut. This state of health was achieved by the interaction between the broiler chicken gut and probiotics. As a result, there was a substantial increase in the weight gain, feed consumption, and FCR. Emerging research on the use of probiotics and prebiotics to enhance the gut health of pigs and poultry has revealed that improved gut health is associated with improved meat quality. These changes manifest as reduced fat content and lean meat [128,129]. Such instances demonstrate that a healthy gut contributes to increased efficiency in nutrient use and digestion, and also provides a pathway to increase growth rate and feed conversion, along with sustainability and the economic returns of animal production.

Nutrient availability and utilization, which directly influence animal performance, are enhanced through modulation of gut health. This can be achieved by administering probiotics and prebiotics. Improved gut health promotes increased efficiency of feed digestion and nutrient absorption, leading to a higher FCR, accelerated growth rates, and enhanced overall feed efficiency [129,130,131]. The enhancement of gut health facilitates physiological adaptation to stressors and environmental challenges. This process subsequently promotes the growth and functional performance of animals. Therefore, an efficient Gut Health Index (GHI) contributes to improved productivity and increased profitability of monogastric livestock production systems. Supplementary Table S2 shows the impact of gut health on the balanced microbiota and the role of probiotics and prebiotics in nutrient digestion and assimilation in monogastric species. It describes the impact on proteins, fats, and carbohydrates as well as on feed conversion, BW gain, and health.

Improved nutrient absorption and reduced nutrient loss at the gut level have system-wide consequences; the next section links enhanced digestive efficiency to reductions in nitrogen and phosphorus excretion and other environmental footprints of swine and poultry production.

## 7. Environmental Implications of Gut Health and Nutritional Efficiency

Sustainability of environmental resources in livestock production systems is critical. Conventional livestock production methods impose substantial ecological pressure, resulting in the pollution of atmospheric and aquatic environments [132,133]. Long-standing concerns associated with animal agriculture include the excretion of nitrogen and phosphorus, which contribute to nutrient pollution, water eutrophication, and the contamination of natural ecosystems. The optimization of gut health in monogastric animals may present a viable strategy to mitigate some of these environmental issues. This can be achieved by improving nutrient absorption and reducing excretion. This bowel extension encourages lean reduction in the environmental impact of livestock agriculture via gut health optimization. This stage prevents the release of nitrogen and phosphorus. It improves the uptake efficiency of dietary nutrients, particularly proteins. Positive outcomes rely on healthy gut microbiota. Enhanced proteolysis and amino acid catabolism resulted in higher protein absorption and lower production of nitrogenous waste. Moreover, healthy gut microbiota modulates the metabolism of other nutrients (e.g., such as carbohydrates and lipids). It also reduces the quantity of undigested food that enters the large bowel. Consequently, the excretion of nitrogen and phosphorus from the waste is diminished [134,135].

Recent reports indicate that gut microbiota-derived SCFAs, especially butyrate, propionate, and acetate, may be key factors related to nutrient metabolism and feed efficiency in animals. These microbial metabolites have dual roles in that they are both energy substrates for colonocytes and modulators of host metabolic pathways that affect nitrogen retention and protein synthesis. The production of SCFAs by microbial fermentation of dietary fibers strengthens the intestinal barrier function by upregulating the expression of tight junction proteins, thus preventing the translocation of undigested food and pathogens into the systemic circulation. The enhanced barrier function directly results in better nutrient retention and less environmental pollution owing to defecation [136,137,138].

50% nitrogen and phosphorus load reduction was observed as a result of the use of probiotics, prebiotics, and synbiotics. For instance, experiments in pigs have shown that supplementation with *Lactobacillus* probiotics reduces nitrogen levels due to improved protein degradation, with a reduction in protein fermentation in the gut. Similarly, the addition of prebiotics, such as oligosaccharides, has a positive influence on gut health and, as a result, feed digestibility is increased, which reduces the amount of undigested feed entering the hindgut and, consequently, the amount of nitrogen and phosphorus excreted [139,140].

Phytase application is also an extremely efficient method to minimize phosphorus loss in monogastric animals. Supplementation with phytase in the range of 500–1500 FTU/kg feed increased the availability of phosphorus through the release of phytate-encapsulated phosphorus in plant-based feed materials, leading to a 27–45% reduction in phosphorus excretion in poultry and swine production systems. This enzymatic approach reduces not only phosphorus pollution in the environment, but also the use of costly inorganic phosphorus sources, which is beneficial for the economy and the environment. Additionally, phytase supplementation increases the availability of other minerals (e.g., as Ca, Zn, and Fe), which are typically bound by phytate, thus enhancing the efficiency of mineral utilization [141,142,143,144,145].

Prebiotics can also favor the growth of certain bacterial species that specialize in nutrient utilization. This selective accumulation in response to influencing factors may greatly reduce environmental pollution. Earlier studies have shown that certain probiotics, including *Bifidobacterium*, decrease ammonia production from manure. These probiotics contribute to the digestion of nitrogenous substances in the gut, leading to a reduced nitrogen output as ammonia. The blockade of fecal nitrogen and phosphorous excretion via a healthier gut provides dietary benefits to animals; meanwhile, it is a major means of reducing the environmental impact of agriculture and animal production [12,146].

Recent data suggest that phytogenic feed additives, such as essential oils (EOs), plant extracts, and secondary plant metabolites, or bioactive botanical substances, may have multiple beneficial effects on gut health and consequently provide indirect benefits for environmental sustainability via environment-friendly ingredients. These natural products improve the barrier function of the gut by increasing the expression of tight junction proteins, including occludin, claudin, and zonula occludens-1 (ZO-1), leading to decreased nutrient leakage and enhanced digestibility. More precisely, phytogenic feed additives with thymol, carvacrol, and other phenolic compounds can shift the gut microbiota composition towards beneficial bacteria such as Lactobacillaceae and reduce potentially pathogenic Enterobacteriaceae. The change in the microbial population is associated with higher protein digestibility and decreased nitrogen excretion, and the mitigated ammonia release from the manure of treated animals has been documented to be in the range of 10–20% [147,148,149,150].

Furthermore, the use of organic acids and medium-chain fatty acids (MCFAs) as additives has been reported as an effective approach for attenuating the environmental impact owing to better utilization of nutrients. Intestinal health is improved by these molecules through pH modulation, eubiosis, and the prevention of pathogen colonization. Reviews indicate that 3–8% improvements in FCR associated with organic acid supplementation have indirect implications of a reduced use of resources and a reduction in environmental burden per unit of animal product. The antibiotic activity of such feed additives also reduces therapeutic antibiotic treatments, and thus concerns related to antibiotic resistance, a very important component in sustainable livestock production [151,152,153,154].

However, improving gut health also decreases emissions of greenhouse gases such as methane and ammonia by reducing nitrogen and phosphorus excretion. Methane is generated during feed fermentation in the digestive tract. This phenomenon is mainly observed in ruminants. Monogastric species are also methane producers, but with smaller amounts and poorer quality. Although the magnitude of methane production is vastly different in animal production, the present study highlights that because the growth of animal production is so great, the effect is nonetheless substantial. In addition, there has been a reduction in methane production from pigs and poultry, which has been ascribed to better gut health and feed efficiency. Probiotics and prebiotics can change the composition balance of the gut microbiota to support the production of less methane as a result of fermentation [12]. Moreover, it has been demonstrated that certain feed additives, such as tannins, can diminish the rate of methane production by downregulating the activity of methanogenic archaea within the gastrointestinal tract. Additionally, improved gut health may contribute to the decreased release of ammonia, a flammable compound that can be emitted from animal feces and is implicated in air pollution. Amino acids are primarily synthesized in the gastrointestinal tract (GIT) of pigs and poultry via protein degradation. The formation of ammonia in manure is directly dependent on the quantity of undigested proteins that reach the large intestine. Hence, interventions for the preservation of optimal gut health, those that support protein digestion, can attenuate ammonia generation to a considerable extent. Strategies to promote gut health in livestock also hold the promise of significantly diminishing methane and ammonia production. These emitters account for a substantial proportion of the environmental burden of livestock production. As a result, these approaches serve the higher-level objective of advancing the sustainability of animal agriculture [155,156].

Life cycle assessment (LCA) research on gut health-based strategies for improving the environmental sustainability of animal production is inclusive. Recent LCA studies of poultry and pig production systems have shown that improving FCE via improvements in gut health, and consequently productivity, reduces the carbon footprint by 8–15% per unit of product. These decreases were attributed to a reduction in feed demand, nutrient excretion, and greenhouse gas (GHG) emissions per animal product unit. Specifically, LCA research reveals that interventions that improve FCR by 0.1 unit through better gut health can reduce GHG emissions by approximately 3–5% along with reductions in land use, water use, and eutrophication potential. The combined use of various gut health approaches, such as probiotics, prebiotics, enzymes, and phytogenic additives, can result in synergistic effects that optimize production efficiency and ecological sustainability [157,158,159,160,161].

In addition to nitrogen, phosphorus, and GHG emissions, there are methods to reduce the environmental burden of meat production. Targeted management of gut health to improve nutrient uptake may also result in decreased feed and other inputs. These advances provide a more sustainable foundation for meat production [135,140]. Therefore, animals are optimal in terms of environmental concerns when they consume less feed and produce more with fewer inputs. Because feed efficiency is a major determinant of livestock production, a better FCR also implies a lower demand for natural resources such as land, water, and energy. Potentially, better nutrient utilization by means of improved gut health may also lead to a decrease in the amount of feces released. If well-managed, this depletion does not significantly harm the environment. Improved gastrointestinal health reduces the number of undigested feed particles in manure. This makes the waste easier to handle. The main regulatory focus is to reduce excess nutrients, such as nitrogen and phosphorus, in the manure. These nutrients are linked to heavy environmental problems, such as leaching into the surrounding water bodies, water contamination, eutrophication, and negative impacts on algal blooms and aquatic life. Therefore, improving feed efficiency, handling waste, and reducing nutrient pollution are areas of focus. Together, these factors facilitate the establishment of an environmentally sustainable animal production system [135,140].

In the pig production system of the circular economy, the role of gut health in nutrient recycling and waste valorization is crucial. Enhanced gut health allows animals to exploit novel feedstuffs, such as by-products of agriculture and food waste, which decreases the pressure on feed resources that arise from humans and the environment. In light of recent developments, it has been demonstrated that gut-healthy animals can cope with up to 30–40% alternative feed ingredients without loss of performance and contribute to closed-loop nutrient cycling systems. This method is in accordance with circular economy policies, as it converts prospective waste streams into high-quality livestock feed materials and conserves superior production efficiency and low environmental impact [162,163,164,165,166].

In addition, precision nutrition approaches based on gut health monitoring and interventional strategies will allow for dynamic tailoring of nutrient supply to the nutritional needs of animals. This nutrient-focused formulation approach reduces nutrient oversupply and excretion from animals, resulting in an estimated reduction of 15–25% of nitrogen excretion and 20–35% of phosphorus excretion compared with traditional feeding regimes. Advanced technologies, such as real-time health monitoring and microbiome profiling, enable customized nutritional management to optimize production efficiency and environmental sustainability [167,168,169].

The husbandry of climate-smart livestock is the cornerstone of improving the environmental sustainability of livestock systems. It will also be key to making these systems more resilient in the face of the challenges of climate change and growing global demand for animal protein. Such gut health-optimizing management practices, for example, the use of feed additives such as probiotics and prebiotics, may result in enhanced nutrient utilization, less waste output, and fewer environmental pollutants. In addition, these interventions provide commercial benefits to livestock producers as a result of reduced feed costs and increased production efficiency, and cost savings due to the reduced use of environmental remediation practices, including the treatment of manure and nutrient management [135,140]. The application of these interventions may also lead to improvements in the overall well-being of the animals. This is accomplished by decreasing the prevalence of digestive system disorders and encouraging better growth performance. The integration of these sustainable approaches into animal production systems offers a viable approach for providing economic and environmental sustainability in the agricultural value chain. Notably, potential conflicts and synergies with other environmental indicators should be evaluated when considering the application of gut health management options in fully sustainable scenarios. Although better gut health is invariably associated with reduced nutrient excretion and increased feeding efficiency, the positive environmental impact is far beyond such direct effects, including less use of antimicrobials, better animal welfare, and improved product quality. Cross-cutting sustainability considerations, such as resource use efficiency, emissions mitigation, biodiversity conservation, and socioeconomic implications, need to be incorporated into holistic evaluation frameworks to adequately assess the value of gut health solutions in sustainable livestock production. In future research, integrated management approaches that mine the potential of trade-offs and co-benefits between the environmental, economic, and social pillars of sustainability should be encouraged [159,170].

Although such environmental advantages are significant, they are constrained by practical, economic, and biological limitations. The challenges, sources of variability, and trade-offs faced by producers and researchers are discussed in Section 8.

## 8. Challenges and Considerations

Numerous strategies have been developed for improving the gastrointestinal health of farm animals. There are many challenges with these processes, and some prevent the widespread adoption of such solutions by farmers. One major problem encountered in the literature is the heterogeneity of the effects of probiotic and prebiotic interventions. This variability can be observed when comparing different species and even different breeds within a species [171,172]. While some probiotic or prebiotic treatments can bring about large beneficial effects on gut health, feed conversion ratios, and production in certain breeds/strains, other treatments may have minimal or modest effects. These disparate responses may be attributed to the genetic background, the starting human gut microbiome composition, and environmental factors. In addition, there are differences in nutritional demands between young and healthy animals and older or sick animals; therefore, nutritional management should be adapted considering these two aspects of physiology and health. Thus, strategies to improve gut health may be more specific. Furthermore, taking into account breed-specific factors may also increase the success of these treatments [173,174].

Gut microbiota composition varies between individual animals, posing a major problem in homogenizing gut health protectants. Recent metagenomic analyses have shown striking variation between individuals in communities of microbes, as well as among animals positive for the same breed, age group, and husbandry systems. This heterogeneity of the microbiome affects responses to probiotics, prebiotics, and other functional feed ingredients, resulting in inconsistent responses among individuals within a production group. Therefore, precision livestock farming strategies that integrate microbiome profiling and tailored nutrition may be required to maximize the intervention efficacy. However, developing these kinds of precision strategies on a commercial scale is still challenging from an economic and logistical perspective, especially for small- or medium-sized agricultural businesses [175,176,177,178].

In addition, the lack of standardized challenge models and response measures to assess interventions for gut health impedes the translation of research into application. Variations in the experimental design, dose regimens, and methods of evaluation of studies prevent direct comparability and the development of evidence-based guidance for producers. A universally accepted definition of “gut health” does not exist, which further complicates the identification of objective markers of intervention success. Standardized methods and protocols, such as FCR, growth performance, and disease incidence, should be used; however, using single parameters to determine gut health is hardly sufficient, as gut health is determined by several biomarkers, such as intestinal morphology, barrier functions, microbial diversity, immune status, and the host’s metabolome [175,179].

The cost associated with probiotics, prebiotics, or other feed additives could be another barrier to the adoption of these strategies in the developing world. Although these additives were found to have some positive effects on nutrient utilization, animal productivity, and environmental management, their application is associated with considerable costs [132,180]. Fermentation, enzymes, probiotics, and prebiotics used in animal feed can also increase the cost of the feed. The economic impact of this could also be a very difficult challenge for producers, especially those in developing countries, as the profit margin on feed is not that wide. In addition, the price of these supplements is not fixed and depends on their dosage, administration frequency, and functional role(s) in the diet. The question is how to recover the cost of developing these products. This is especially true in cases where the benefits of their use cannot be easily measured in the short term. In addition, such benefits may not be directly reflected on the top line. Such financial considerations can be challenging for cattlemen. This is especially true when there are cheaper and more readily available feeding alternatives [174].

The cost–benefit evaluation of gut health strategies should also consider benefits that are not directly related to production, such as improved animal welfare, reduced antimicrobial use, and better environmental sustainability. However, although probiotics and prebiotics may increase feed costs by 3–8%, their use can reduce expenses for veterinary services by lowering the incidence of disease, death, and use of therapeutics. Furthermore, the economic benefits of enhanced animal welfare are now appreciated by consumers and the legislation. These benefits might include lower stress behavior, increased immune activity, and greater tolerance to environmental stressors, and may allow a price premium for products from animals produced in welfare-enhanced systems. Economic evaluations that include these multiple benefits from a long-term perspective need to be undertaken to accurately assess the commercial feasibility of strategies to manipulate gut health [7,181,182,183,184].

However, as noted above, regulatory and market pressures continue to play pivotal roles in the development of probiotics and prebiotics for use in animal production. Regulations for feed additives are different in each country or region. There are also often legal provisions under which such products must be licensed and approved in various countries, and which harmonization in the sense of “permission to use” in industrial animal feed may be sought for the first time together with certification. These challenges slow down the development and delivery of effective gut health strategies, delaying their adoption and increasing costs. Consumer acceptance remains a major challenge. Even though animal feed may be off the table for people who like to eat clean or organic food, any products that contain probiotics or prebiotics, routine ingredients in human nutrition and health products, may not fare as well with animal feed consumers, assuming they have a strong opinion on the matter. The impact of these compounds on the quality and safety of foods of animal origin is unknown. Furthermore, consumers’ perceptions of these additives can influence the market and industry. A key to promoting a more holistic view of the impact of probiotics and prebiotics at the societal level is to develop means to increase consumers’ familiarity with the subject. These policies should also consider the implications for animal welfare and environmental consequences.

Studies on consumer perceptions reveal complex and sometimes inconsistent positions regarding the use of functional feed additives in the production of meat. Although consumers appeared to be positive for the majority of products designed to improve animal welfare and reduce antibiotic usage in the market, skepticism was evident around the “naturality” of probiotic supplementation, especially from organic food consumers who believed that natural additives are not suitable for use in conventional agriculture. Openness in the production process, clear labeling, and consumer awareness campaigns are essential to address these concerns and gain trust in interventions in gut health as an example of enhancing welfare. Studies have shown that consumer acceptance, when informed about the modes through which probiotics contribute to better animal health, such as lower incidence of disease, improved tolerance to stress, and reduced use of therapeutic antibiotics, can be far higher. In this context, 72–92% would be willing to buy animal products from animals fed functional additives [184,185,186,187].

The potential animal welfare effects of optimizing gut health may, therefore, be considered to include the behavioral and psychological well-being associated with it. Recent reports have shown that the composition of the gut microbiota not only plays a role in the regulation of digestive and immune functions but is also involved in adaptation to stress, the behavior of animals, and the general state of animal welfare in the livestock industry. Gut dysbiosis has been linked to the increased expression of abnormal behaviors, elevated stress responses, and diminished capacity for environmental adaptation. In contrast, treatments that encourage beneficial gut microbiota, such as probiotics, prebiotics, and good nutrition, have demonstrated positive effects on the reduction of stress-related behaviors, improvement of social interactions, and increased resistance to management difficulties, such as weaning, transport, and climatic environmental changes. Fecal microbiota transplantation (FMT) is a novel therapeutic strategy that has the potential to quickly reconstitute the healthy gut microbiota of animals with dysbiosis. The reported advantages of FMT are better production performance, lower incidence of disease, fewer abnormal behaviors, and better stress coping [183,188].

Another example of the gut health-animal welfare association is the gut–brain axis, a dual communication system between the gastrointestinal microbiota and the central nervous system through neural, endocrine, and immune pathways. Gut microbial metabolites, notably SCFA, modulate brain activity, neurotransmitter production, and the host’s behavioral responses. Hence, enhancing gut health via specific dietary measures could improve physical health as well as cognitive performance, mood, and behavior, all of which are imperative for an animal’s welfare as a whole. This holistic view elevates the management of gut health to a foundational element in welfare-based livestock systems that focus on the quality of life and production efficiency [6,136,189].

In addition to animal welfare improvements, there is a welfare benefit that can affect humans by decreasing the buildup of antimicrobial resistance, a major concern for both animal and public health, as a result of reduced antimicrobial usage and enhanced gut health. Animals with healthy guts that are raised in nutritional regimens geared toward disease prevention are less challenged by infectious diseases, require less treatment, have stronger immune functions, and in turn, reduce the risk of welfare problems. The shift from reactive antibiotic treatment to proactive management of gut health is consistent with current animal welfare models. Such models prioritize prevention rather than treatment and understand that genuine welfare includes physical health, psychological well-being, and natural behavior [6,152,153,182,190].

Future research should aim to construct integrated evaluation paradigms that consider production efficiency, environmental sustainability, and animal welfare implications of gut health management. Longitudinal research on animal life stages and production systems is required to comprehensively assess the enduring effects of a range of gut health methodologies on animal welfare, behavior, and robustness. In addition, this discussion highlights the need for increased interdisciplinary cooperation among animal nutritionists, veterinarians, microbiologists, ethologists, and welfare scientists to develop more comprehensive solutions for sustainable livestock production. These strategies consider the gut as a key influencer of animal welfare, production, and sustainability [175,177,191].

Overcoming these challenges will require appropriate research and precision strategies. The following section presents key research topics that will facilitate the upscaling and strengthening of gut health-based approaches.

## 9. Future Directions and Research Gaps

Future work needs to transcend known restating mechanisms to fill actionable knowledge gaps that allow for focused, stewardship-based translation. Goals are: (i) integration of multi-omics (metagenomics, metatranscriptomics, metabolomics) with longitudinal host readouts to link microbial activities with barrier, immune and performance endpoints to define causality; (ii) design and validation of pre-dictive biomarkers and decision-support tools that segregate animals according to their probability to respond in a way that allows for precision nutrition aligned to genotype, microbiome profile, health status, and diet matrix; (iii) rational design of synbiotics and tailored prebiotic–probiotic formulations with evidence of substrate degradation and functional responses in pigs and poultry; (iv) evaluation of innovative, scientific supported additives—such as plant-derived bioactives [EO, polyphenols], seaweeds, fermented matrices—with sound head-to-head comparisons to traditional ones; (v) translational trials at commercial level with harmonized outcome reporting extending from performance and welfare indicators, to well-defined antimicrobial use to environmental surrogates (e.g., manure N/P, NH_3_), comprehensively including cost-effectiveness and simple life-cycle assessing; and (vi) enhance reproducibility by preregistration, good power, transparent statis-tical reporting, and open data. Functional annotation of microbiomes, validation of biomarker panels for use in the field, tools for personalized feeding, and thorough assessment of additive interplay (synergy and redundancy) remain important gaps. Filling in these gaps will allow more accurate fine-tuning of gut health, reduce dependence on antibiotics, limit waste, and increase both production and environmental sustainability in monogastric animal systems. By combining mechanistic understanding with farm-level application—tailored nutrition, diagnostics, and multi-disciplinary science—gut health solutions will provide the means to achieve concurrent welfare, productivity, and environmental sustainability.

## 10. Conclusions

Gut health management plays a vital role in the welfare, productivity, and environmental sustainability of monogastric livestock. Nutritional strategies, including probiotics, prebiotics, and other feed ingredients, play an important role in modulating gastrointestinal function and the immune response. They might help decrease dependence on therapeutic and prophylactic antibiotics, aligning with antibiotic stewardship in animal production. These interventions positively affect the gut microbiota by increasing the relative abundance of commensal bacteria (such as *Lactobacillus* and *Bifidobacterium*), enhancing microbial richness and stability, and reducing pathogen contamination. These alterations lead to an enhanced mucosal barrier and higher SCFA levels, which in turn promote more efficient nutrient uptake. Thus, they play a role in enhancing growth rates and FCRs, as well as in reducing waste production and environmental pollution. Hopefully, these mindsets will be carried forward in the pursuit of R&D (and not merely sustainability in agri-sector research). In particular, in life cycle management, these strategies can serve to enhance the sustainability of the industry and potentially reduce its environmental impact.

## Figures and Tables

**Table 1:** Summary of key dietary nutrients and functional feed additives that enhance gut health, modulate microbiota, and improve performance in monogastric livestock.

Nutrient/Additive	Type	Source/Examples	Function/Role	Effect on Gut Health	Impact on Livestock Performance	Potential Risks/Considerations	References
Dietary Fibers	Non-starch polysaccharides (NSPs)	Plant-based fibers (e.g., cellulose, hemicellulose, oats, barley)	Stimulate bacterial growth, provide energy for gut microbiota, enhance digestive processes	Improve gut microbiota balance, increase bacterial diversity, and enhance nutrient absorption	Enhanced digestion, improved nutrient absorption, increased weight gain	Excessive fiber may reduce energy intake or cause digestive discomfort	[50,51]
Amino Acids	Essential and non-essential	Animal and plant-based proteins (e.g., lysine, methionine, soy, fishmeal)	Essential for protein synthesis, immune response, and gut tissue repair	Support immune function, gut tissue regeneration, and enhance intestinal health	Improved growth rates, stronger immune response	Imbalanced amino acid profiles can affect growth and health	[52,53]
Antioxidants	Vitamins A, C, E	Fruits, vegetables, animal products (e.g., liver, carrots)	Protect gastrointestinal lining from oxidative stress and inflammation	Protect gut lining from damage, reduce inflammation, and enhance gut barrier function	Improved immunity, reduced incidence of gastrointestinal diseases	Over-supplementation may disrupt nutrient absorption or cause toxicity	[54,55]
Prebiotics	Oligosaccharides, Inulin	Plant fibers, legumes (e.g., chicory, garlic)	Promote the growth of beneficial bacteria by providing a food source for them	Enhance gut microbiota composition, strengthen intestinal barrier, improve gut immune response	Better nutrient absorption, improved growth, and feed conversion	Overfeeding may cause fermentation imbalances or gastrointestinal upset	[56,57]
Probiotics	Live beneficial bacteria	Fermented foods, supplements (e.g., *Lactobacillus*, *Bifidobacterium*)	Introduce beneficial microbes to balance gut microbiota, suppress harmful bacteria	Restore microbiota balance, improve digestion, suppress pathogenic microorganisms	Improved feed conversion, enhanced immune function, reduced disease incidence	Probiotics must be stored correctly; incorrect strain may not have beneficial effects	[54,55]
Synbiotics	Combination of prebiotics and probiotics	Combination of dietary fibers and live beneficial bacteria	Support gut health by combining the benefits of both prebiotics and probiotics	Improve gut microbiome, boost immunity, enhance feed conversion efficiency	Increased growth, enhanced immune function, better feed utilization	Strain and dosage must be appropriately matched for effectiveness	[54,55]
Short-Chain Fatty Acids (SCFAs)	Acetic, propionic, butyric acids	Fermentation products (from fiber)	Provide energy to colonocytes, modulate immune responses, lower gut pH to inhibit pathogenic bacteria growth	Support gut epithelial health, reduce inflammation, promote beneficial microbial growth	Improved intestinal health, reduced gastrointestinal distress, enhanced growth	Overproduction of SCFAs could lower gut pH excessively, inhibiting absorption	[56,57]
Polyphenols and Flavonoids	Plant-derived compounds	Fruits, vegetables, herbs (e.g., blueberries, apples, green tea)	Possess antimicrobial, antioxidant, and anti-inflammatory properties	Decrease oxidative stress, modulate gut microbiota, improve gut health by strengthening the gut lining	Reduced oxidative stress, better gut lining integrity, improved immune function	High doses may cause digestive upset or interfere with mineral absorption	[56,57]

## Data Availability

No new data were created or analyzed in this study. Data sharing is not applicable to this article.

## Supplementary Materials

The following supporting information can be downloaded at: https://www.mdpi.com/article/10.3390/vetsci12111054/s1, Table S1: Probiotics and Prebiotics: Mechanisms of Action for Gut Health in Monogastric Livestock. Table S2: Impact of Gut Health on Nutrient Absorption and Utilization in Monogastric Livestock [19,48,70,79,100,101,102,103,104,105,106,108,109,111,120,121,128,130,131,192,193,194,195,196,197,198].
