# Supplementary material for: Probiotics, Prebiotics, and Synbiotics in Pigs and Poultry: A Review of Gut Health, Performance, and Environmental Outcomes
Source: Vet Sci. 2025 Nov 2;12(11):1054. doi: 10.3390/vetsci12111054 (PMC12656940; doi:10.3390/vetsci12111054)
Supplement: Supplementary file 1 [file vetsci-12-01054-s001.zip › vetsci-3910656-supplementary.pdf]

Supplemental Table S1: Probiotics and Prebiotics: Mechanisms of Action for Gut Health in Monogastric Livestock

Component	Type	Source/Examples	Mechanism of Action	Effect on Gut Health	Impact on Livestock Performance	Potential Risks/Considerations	References
**Probiotics**	Viable, non-pathogenic microbes	Lactobacillus, Bifidobacterium species, yeast (e.g., Saccharomyces boulardii)	Enhance the growth of beneficial bacteria, compete with pathogens for space and resources, release antimicrobial compounds (e.g., lactic acid, bacteriocins)	Maintain gut microbiota balance, improve intestinal barrier integrity, stimulate mucus production, reduce pathogen load	Improved growth rate, nutrient utilization, immune response, and disease resistance	Misuse or over-supplementation could disrupt the natural microbiota or cause imbalances	[192]
**Lactobacillus Species**	Lactic acid bacteria	Fermented foods, supplements	Produce lactic acid to maintain acidic pH in the gut, inhibit growth of harmful bacteria, promote SCFAs production	Lower gut pH, inhibit pathogenic bacteria, stimulate gut health by producing SCFAs (e.g., butyric acid)	Enhanced growth, better feed conversion, reduced pathogenic bacteria, improved immunity	Some strains may not survive in the gastrointestinal tract under certain conditions	[193]
**Bifidobacterium Species**	Beneficial bacteria	Fermented products, supplements	Metabolize carbohydrates into SCFAs, enhance growth of beneficial bacteria, and modulate immune responses	Improve gut barrier function, increase beneficial microbiota, support immune system function	Better growth, enhanced feed conversion, and immune system improvement	Can cause digestive upset if administered in excess	[194]

**Prebiotics**	Non-digestible food ingredients	Oligosaccharides, Inulin, Fructooligosaccharides (FOS)	Serve as a food source for beneficial bacteria, selectively enhance the growth of good bacteria, and inhibit pathogen growth	Improve gut microbiota composition, increase SCFAs production, lower gut pH, and enhance gut barrier function	Better digestion, nutrient absorption, improved immune function, enhanced productivity	High doses may cause digestive discomfort, bloating, or flatulence	[195]
**Oligosaccharides**	Short-chain sugar molecules	Legumes, beans, chicory, garlic, onions	Not digestible in upper GI tract, reach colon where they are fermented by beneficial bacteria	Stimulate growth of Bifidobacterium, increase SCFAs, reduce gut pH, and inhibit pathogenic bacteria	Enhanced nutrient absorption, improved feed conversion, reduced disease susceptibility	May cause discomfort in high quantities or alter gut microbiota composition	[196]
**Inulin**	Fructan polysaccharide	Chicory, Jerusalem artichokes, garlic, onion	Stimulates Bifidobacterium growth, increases SCFAs levels, and reduces gut pH to limit pathogen growth	Increase in beneficial gut bacteria, improved gut barrier function, enhanced digestion, and immunity	Improved nutrient utilization, enhanced growth rates, and better feed conversion	It can cause bloating or flatulence when administered in excess	197]
**Synbiotics**	A combination of probiotics and prebiotics	Combination of probiotics (e.g., Lactobacillus, Bifidobacterium) and prebiotics (e.g., FOS, inulin)	Provide both beneficial microorganisms and food sources for these microbes, enhancing the synbiotic relationship	Improve gut microbiota, enhance digestion, stimulate immune response, and reinforce gut barrier integrity	Improved growth, nutrient absorption, better immunity, and reduced antibiotic use	Strain/dose mismatch can limit effectiveness; may cause gastrointestinal upset	[79]

**Overall Effects of Synbiotics**	Combined effect of probiotics and prebiotics	A combination of various beneficial probiotics and prebiotics	Enhance gut health synergistically by combining the benefits of both probiotics and prebiotics, improving overall gut function	Balanced gut microbiome, reduced pathogenic microorganisms, better nutrient absorption, enhanced immune function	Increased productivity, improved growth rates, better feed conversion, reduced antibiotic usage	May have varying effects based on livestock species, strain, and diet composition	[70,198]

Supplementary Table S2: Impact of Gut Health on Nutrient Absorption and Utilization in Monogastric Livestock

Factor	Role of Gut Health/Probiotics/Prebiotics	Mechanism of Action	Impact on Nutrient Utilization	Effect on Animal Performance	Key Studies/Findings
**Gut Microbiota**	Natural gut flora of monogastric animals	Breaks down larger nutrient particles into smaller molecules, releases SCFAs like butyrate, and supports the function of nutrient transporter genes	Enhances protein, fat, and carbohydrate digestion and absorption through improved microbial activity	Improved nutrient solubility, absorption, and energy utilization for metabolism and growth	[100,101]
**Protein Metabolism**	Probiotics and prebiotics (e.g., Lactobacillus, Bifidobacterium)	Enhances the breakdown of proteins into peptides and amino acids, and increases proteolytic enzyme production by beneficial bacteria	Improved protein digestion and amino acid absorption, aiding in muscle growth and tissue accretion	Increased growth rates, better muscle development, improved feed conversion ratio (FCR)	[101,103, 104]
**Fat Metabolism**	Probiotics (Lactobacillus, Bifidobacterium)	Microbial fermentation of undigested carbohydrates to produce SCFAs, which aid in fat metabolism and reduce fat accumulation	Improved lipid digestion, increased fat utilization for energy, and better body composition	Enhanced fat utilization for energy production, better body composition, and increased energy availability for growth	[104,105,106]

**Carbohydrate Metabolism**	Probiotics and prebiotics (e.g., inulin, oligosaccharides)	Microbial breakdown of complex carbohydrates into simple sugars that can be absorbed by the gut cells	Increased digestion of carbohydrates, improved glucose absorption, and energy utilization	Improved feed intake, increased growth rates, and better feed conversion	[101,102,103]
**Gut Barrier Function**	Probiotics (e.g., Lactobacillus, Bifidobacterium)	Stimulate mucus and tight junction protein production, strengthen the epithelial barrier to pathogens and toxins	Increased intestinal barrier integrity, reduced pathogen invasion, and inflammation	Improved disease resistance, reduced incidence of gastrointestinal diseases (e.g., diarrhea), better growth, and feed conversion	[120,121]
**Feed Conversion Efficiency**	Probiotics, prebiotics (e.g., inulin, oligosaccharides)	Increased nutrient solubility and absorption through enhanced gut microbial activity	More efficient nutrient digestion and absorption, improved feed conversion ratio (FCR)	Increased growth rate, less feed required for weight gain, reduced feed costs	[111]
**Growth Rate**	Probiotics, prebiotics, synbiotics	Enhanced nutrient absorption, improved protein, fat, and carbohydrate metabolism, reduction of gastrointestinal disorders	Accelerated weight gain, increased muscle growth, improved overall productivity	Higher weight gain, improved growth performance, reduced time to market	[108,109]
**Stress Tolerance**	Probiotics and gut microbiota stability	Modulation of immune responses, reduction of cortisol (stress hormone), control of oxidative stress and inflammation	Reduced negative impact of stress on digestion and growth, improved immune response	Improved health, better growth rates, enhanced ability to tolerate environmental stress, improved feed intake, and FCR	[48,101]
**Disease Resistance**	Probiotics and prebiotics (e.g., Lactobacillus, Bifidobacterium)	Strengthen gut microbiota, enhance gut barrier function, improve immune system responses	Reduced pathogen load, improved immune system activity, and reduced gastrointestinal diseases	Better health, fewer diseases, improved feed conversion, better weight gain, reduced need for antibiotics	[18,19]
**Meat Quality**	Probiotics and prebiotics	Improve nutrient digestion and	Enhanced lean meat yield, improved quality	Increased quality and yield of lean meat,	[128]

		absorption, reduce fat content, and increase lean meat yield	of meat due to better nutrient utilization	better fat-to-muscle ratio in pigs and poultry	
**Overall Productivity**	Probiotics, prebiotics, synbiotics	Optimized gut health leads to better nutrient utilization, reduced gastrointestinal disorders, and enhanced immune function	Increased feed efficiency, faster growth rates, improved overall performance	Improved productivity, reduced feed costs, and better profitability in livestock production	[130,131]

References

19. Jha, R.; Fouhse, J.M.; Tiwari, U.P.; Li, L.; Willing, B.P. Dietary Fiber and Intestinal Health of Monogastric Animals. Front. Vet. Sci. 2019, 6, 48. https://doi.org/10.3389/fvets.2019.00048.

48. Wang, Y.; Wu, Y.; Chen, J.; Guo, X.; Yan, L.; Guo, Y.; Wang, B.; Yuan, J. The Duration of Food Withdrawal Affects the Intestinal Structure, Nutrients Absorption, and Utilization in Broiler Chicken. FASEB J. 2021, 35, e21178. https://doi.org/10.1096/fj.202001773R.

70. Yue, T.; Lu, Y.; Ding, W.; Xu, B.; Zhang, C.; Li, L.; Jian, F.; Huang, S. The Role of Probiotics, Prebiotics, Synbiotics, and Postbiotics in Livestock and Poultry Gut Health: A Review. *Metabolites* **2025**, *15*, 478, doi:10.3390/metabo15070478.

79. Markowiak, P.; Śliżewska, K. Effects of Probiotics, Prebiotics, and Synbiotics on Human Health. *Nutrients* **2017**, *9*, 1021, doi:10.3390/nu9091021.

100. Zhang, H.; Zheng, T.; Wang, Y.; Li, T.; Chi, Q. Multifaceted Impacts of Nanoparticles on Plant Nutrient Absorption and Soil Microbial Communities. *Front. Plant Sci.* **2024**, *15*, doi:10.3389/fpls.2024.1497006.

101. Yadav, S.; Jha, R. Strategies to Modulate the Intestinal Microbiota and Their Effects on Nutrient Utilization, Performance, and Health of Poultry. *Journal of Animal Science and Biotechnology* **2019**, *10*, 2, doi:10.1186/s40104-018-0310-9.

102. Xu, P.; Gao, Y.; Cui, Z.; Wu, B.; Yan, B.; Wang, Y.; Zaitongguli, K.; Wen, M.; Wang, H.; Jing, N. Research Progress on Effects of Biochar on Soil Environment and Crop Nutrient Absorption and Utilization. Sustainability 2023, 15, 4861.

103. Singh, A.K.; Kim, W.K. Effects of Dietary Fiber on Nutrients Utilization and Gut Health of Poultry: A Review of Challenges and Opportunities. *Animals* **2021**, *11*, 181.

104. Ravindran, V.; Kornegay, E.T.; Webb Jr, K.E. Effects of Fiber and Virginiamycin on Nutrient Absorption, Nutrient Retention and Rate of Passage in Growing Swine. *Journal of Animal Science* **1984**, *59*, 400–408.

105. Rani, R.P.; Nishant, A.K.; Kumar, G. Nutrient utilization and crop metabolism. Crop Physiol. A Collab. Insights 2023, 86.

106 Gilbert, E.R.; Wong, E.A.; Webb Jr, K.E. Board-Invited Review: Peptide Absorption and Utilization: Implications for Animal Nutrition and Health. *J. Anim. Sci.* **2008**, *86*, 2135–2155.

108. Trovato, A.; Nuhlicek, D.N.; Midtling, J.E. Drug-Nutrient Interactions. Am. Fam. Physician 1991, 44, 1651–1658.

109. Selim, S.; Abdel-Megeid, N.S.; Khalifa, H.K.; Fakiha, K.G.; Majrashi, K.A.; Hussein, E. Efficacy of Various Feed Additives on Performance, Nutrient Digestibility, Bone Quality, Blood Constituents, and Phosphorus Absorption and Utilization of Broiler Chickens Fed Low Phosphorus Diet. Animals 2022, 12, 1742.
111. Piantoni, P.; VandeHaar, M.J. Symposium Review: The Impact of Absorbed Nutrients on Energy Partitioning throughout Lactation. J. Dairy Sci. 2023, 106, 2167–2180.
120. Jia, R.; Sadiq, F.A.; Liu, W.; Cao, L.; Shen, Z. Protective Effects of Bacillus Subtilis ASAG 216 on Growth Performance, Antioxidant Capacity, Gut Microbiota and Tissues Residues of Weaned Piglets Fed Deoxynivalenol Contaminated Diets. Food Chem. Toxicol. 2021, 148, 111962.
121. Fouhse, J.M.; Zijlstra, R.T.; Willing, B.P. The Role of Gut Microbiota in the Health and Disease of Pigs. Anim. Front. 2016, 6, 30–36.
128. van Gennep, S.; Gielen, M.E.; Rietdijk, S.T.; de Boer, N.K.; Duijvestein, M.; Gecse, K.B.; Ponsioen, C.Y.; D'Haens, G.R.; de Boer, A.G.; Löwenberg, M. Work Productivity Loss Is Determined by Fatigue and Reduced Quality of Life in Employed Inflammatory Bowel Disease Patients: A Prospective Multicentre Cohort Study. Eur. J. Gastroenterol. Hepatol. 2021, 33, e594–e602.
130. Loeppke, R.; Taitel, M.; Haufle, V.; Parry, T.; Kessler, R.C.; Jinnett, K. Health and Productivity as a Business Strategy: A Multiemployer Study. J. Occup. Environ. Med. 2009, 51, 411–428.
131. Lopez-Santamarina, A.; Mondragon, A.d.C.; Cardelle-Cobas, A.; Santos, E.M.; Porto-Arias, J.J.; Cepeda, A.; Miranda, J.M. Effects of Unconventional Work and Shift Work on the Human Gut Microbiota and the Potential of Probiotics to Restore Dysbiosis. Nutrients 2023, 15, 3070.
192. Vinayamohan, P.; Joseph, D.; Viju, L.S.; Baskaran, S.A.; Venkitanarayanan, K. Efficacy of Probiotics in Reducing Pathogenic Potential of Infectious Agents. *Fermentation* **2024**, *10*, 599, doi:10.3390/fermentation10120599.
193. Dempsey, E.; Corr, S.C. Lactobacillus Spp. for Gastrointestinal Health: Current and Future Perspectives. *Front Immunol* **2022**, *13*, 840245, doi:10.3389/fimmu.2022.840245.
194. Jena, R.; Choudhury, P.K. coods: A Health Beneficial Outlook. *Probiotics Antimicrob Proteins* **2025**, *17*, 1–22, doi:10.1007/s12602-023-10189-w.
195. Victoria Obayomi, O.; Folakemi Olaniran, A.; Olugbemiga Owa, S. Unveiling the Role of Functional Foods with Emphasis on Prebiotics and Probiotics in Human Health: A Review. *Journal of Functional Foods* **2024**, *119*, 106337, doi:10.1016/j.jff.2024.106337.
196. Jovanovic-Malinovska, R.; Kuzmanova, S.; Winkelhausen, E. Oligosaccharide Profile in Fruits and Vegetables as Sources of Prebiotics and Functional Foods. *International Journal of Food Properties* **2014**, *17*, 949–965, doi:10.1080/10942912.2012.680221.
197. Hughes, R.L.; Alvarado, D.A.; Swanson, K.S.; Holscher, H.D. The Prebiotic Potential of Inulin-Type Fructans: A Systematic Review. *Adv Nutr* **2021**, *13*, 492–529, doi:10.1093/advances/nmab119.
198. Parhi, P.; Liu, S.Q.; Choo, W.S. Synbiotics: Effects of Prebiotics on the Growth and Viability of Probiotics in Food Matrices. *Bioactive Carbohydrates and Dietary Fibre* **2024**, *32*, 100462, doi:10.1016/j.bcdf.2024.100462.
